# Involvement of striatal motoric subregions in familial frontotemporal dementia with parkinsonism harboring the C9orf72 repeat expansions

**DOI:** 10.1038/s41531-022-00398-5

**Published:** 2022-10-06

**Authors:** Li Liu, Shuying Liu, Min Chu, Jingjuan Wang, Kexin Xie, Yue Cui, Jinghong Ma, Haitian Nan, Chunlei Cui, Hongwen Qiao, Pedro Rosa-Neto, Piu Chan, Liyong Wu

**Affiliations:** 1 Department of Neurology, Xuanwu Hospital, Capital Medical University, Beijing, China; 2 Department of Neurology, Shenyang Fifth People Hospital, Shenyang, China; 3 Department of Nuclear Medicine, Xuanwu Hospital, Capital Medical University, Beijing, China; 4 McGill Centre for Studies in Aging, Alzheimer’s Disease Research Unit, Montreal, H4H 1R3 Canada; 5 National Clinical Research Center for Geriatric Diseases, Beijing, China

**Keywords:** Neurodegenerative diseases, Movement disorders

## Abstract

The chromosome 9 open reading frame 72 (C9ORF72) has been proposed as the causative gene of frontotemporal dementia with parkinsonism (FTDP), but its pathophysiological mechanism of parkinsonism is poorly understood. To explore the roles of striatal motor subdivisions in the pathogenesis of parkinsonism resulting from C9ORF72 repeat expansions in the FTDP, two patients with FTDP from one pedigree and seventeen healthy controls were enrolled. The participants received clinical interviews, physical examinations, genetic testing, [^18^F]-fluorodeoxyglucose PET/MRI, and [^18^F]-dihydrotetrabenazine PET/CT. Voxel-wise and region of interest analysis were conducted with respect to gray matter volume, metabolism, and dopamine transport function between patients and controls, focusing on the motor part of the striatum according to the Oxford-GSK-Imanova Striatal Connectivity Atlas. Patient 1 presented with parkinsonism as the initial symptom, while patient 2 exhibited behavior disturbance as the first symptom, followed by parkinsonism within one year. Both patients had the hexanucleotide expansion detected in C9ORF72(>52 repeats). Gray matter volume atrophy, hypometabolism and dopamine dysfunction were observed in the motor areas of the striatum. Of the two patients, marked glucose hypometabolism within the striatal motor subregion was observed in patient 1, with corresponding gray matter atrophy. In addition, presynaptic dopaminergic integrity of patient 2 was deteriorated in the motor subregions which was consistent with gray matter atrophy. These findings imply that parkinsonism in FTDP may be associated with the degeneration and dopaminergic dysfunction of the striatal motor subregion, which might be attributed to C9orf72 repeat expansions.

## Introduction

The chromosome 9 open reading frame 72 (C9orf72) hexanucleotide repeat expansion was first discovered in 2011, which is mainly associated with amyotrophic lateral sclerosis (ALS) and/or frontotemporal dementia (FTD), and is characteristic of involvement in the extramotor neocortex, hippocampus, and lower motor neurons^1–3^. Recent studies have also revealed C9ofr72 as a causative gene of frontotemporal dementia with parkinsonism (FTDP)^4,5^. However, the underlying pathogenesis of parkinsonism in FTDP with C9orf72 repeat expansion remains unclear.

Atrophy, hypermetabolism, and dopaminergic dysfunction with a predominant rostral-caudal gradient in the striatum had firmly been confirmed in Parkinson’s disease (PD)^6–8^. Few research studies in FTDP patients with C9orf72 mutation have revealed the involvement of the striatum based on neuropathological and imaging analysis^9–11^, however, it is ambiguous whether the pattern of striatum involvement is different between C9orf72-related FTDP and Parkinson’s disease. It is worth noting that the striatum has been involved in multiple functions, including motor control, motivated behavior, social emotion, and cognition, and is widely integrated with motor, limbic, associative networks^12–14^. Furthermore, previous studies have only analyzed the striatal lesions in the entire striatum or only made an anatomical dissociation between the caudate nucleus and the putamen, which might not provide optimal parcellation of the striatum to focus on the motor functions. Recently, striatal subregions associated with movement can be analyzed by an atlas based on their distinct cortical connectivity profiles, which functionally defined striatal subregions^15^. In our previous study, we described the first Chinese FTDP family with C9orf72 repeat expansions, however, it is currently unclear whether C9ORF721 mutation carriers develop parkinsonism because of C9ORF721 causing involvement of the striatal motor regions^5^.

In the current study, we conducted a series of assessments in gray matter (GM) volume, metabolism and dopamine transport function in two patients with FTDP who belonged to one family with C9orf72 mutations, focusing on the motor regions of the striatum. The aim was to investigate the alteration in striatal motor subregions associated with parkinsonism in FTDP patients harboring C9orf72 repeat expansions. We hypothesized that C9orf72 repeat expansions might cause impairment of the motor subregion in the striatum, which contributes to the manifestation of parkinsonism in FTDP.

## Results

### Clinical study

**Fig. 1:**
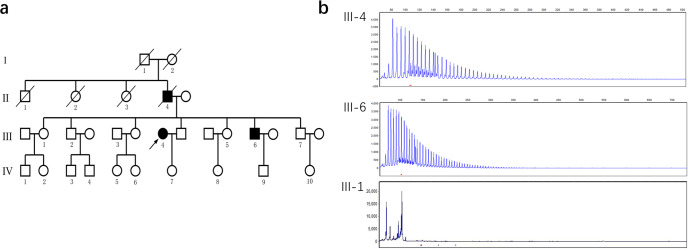
FTDP pedigree with C9ORF72 repeat expansions. **a** Pedigree tree of the family with the repeat expansion mutation in C9orf72. There were three symptomatic patients in four generations of this pedigree. **b** Results of repeat primed PCR for C9orf72 expansion demonstrating the saw-tooth pattern, typical of the pathological expansion. Repeats are measurable up to 52 hexanucleotide repeats. Both the proband (III-4) and her brother (III-6) carry the C9orf72 repeat expansion. The proband’s sister (III-1) has a normal C9orf72 genotype (2/8) and is alive and healthy at the age of 80 years.

We studied a Chinese family in which two living individuals (one female and one male) were affected with FTDP (Fig. 1, Supplementary Table 1). Both patients were examined by brain [^18^F]-FDG PET/MRI, while only patient underwent [^18^F]-DTBZ PET/CT.

### Patient 1 (III-4)

The proband was a 68-year-old woman who developed movement disorders followed by cognitive impairment over a period of three years prior to evaluation. She was referred due to asymmetric parkinsonism with akinesia and rigidity in the upper right limb, and masked facies with a reduced rate of blinking at the age of 65 years. At the same time, the family observed personality changes characterized by dampened emotions, reduced interest in family business matters, and lack of motivation. She was initially misdiagnosed with Parkinson s disease. Levodopa produced a moderated and transient benefit. A year later, she showed marked cognitive impairment involving memory, attention, and executive domains. Progressively, she exhibited generalized bradykinesia and stiffness in the muscles of all limbs and the neck. Three years after symptom onset, she became wheelchair-bound, and responded poorly to levodopa treatment. Consistently, she did not show any signs of amyotrophic lateral sclerosis, including fasciculations, muscle weakness, muscle atrophy, or the Babinski sign. Neuropsychological tests revealed an MMSE score of 13/30, a MoCA score of 5/30, and a global CDR score of 2. The score on UPDRS-III score was 76. Brain MRI showed bilateral and symmetrical anterior frontal and temporal atrophy, but the preservation of the parietal and occipital lobes. Given the clinical, neuropsychological, and imaging findings, the patient received a formal diagnosis of FTDP from three neurologists, three years after the onset of his symptoms.

### Patient 2 (III-6)

Patient 2 (III-6, brother of the proband) presented with behavioral and personality changes at the age of 62 years (Fig. 1). His wife described him as becoming socially withdrawn, increasingly depressed, apathetic, irritable, restless, disinhibited, and disinterested in his family. Less than a year after symptom onset, he developed parkinsonian features such as bradykinesia, symmetric rigidity, and stooped posture and had recurrent falls. Further, he presented progressive memory loss, started to get lost in new surroundings, and displayed impairment in daily activities. At that time, he was diagnosed with frontotemporal dementia. As the disease progressed, he exhibited language difficulties characterized by word-retrieval difficulties and mildly impaired comprehension that later evolved to mutism. There was severe rigidity of the limb muscles without tremor. He visited our clinic three years after the onset of symptoms. Neurological examinations did not reveal fasciculations, muscle weakness, muscle atrophy, or the Babinski sign. Unfortunately, it was difficult to perform a formal neuropsychological evaluation due to severe rigidity of the limbs and aphasia. The UPDRS-III score was 88. Brain MRI showed generalized cortical and cerebellar atrophy most marked in the frontal and temporal regions. Combined the symptoms of abnormal personality -behavior, parkinsonism, and the MRI findings, this patient met the diagnostic criteria for FTDP.

### Other relative (II-4)

According to relatives, the father of the proband and Patient 2 experienced behavioral and personality changes at the age of 72 years. He became restless, disinhibited, and acted in a socially inappropriate manner. He eventually died at the age of 76 years.

### Genetic findings

DNA was available for analysis in the two examined subjects, each of whom had the hexanucleotide expansion detected in C9orf72(>52 repeats). No mutation was present in the other genes associated with FTD, parkinsonism and other neurodegenerative diseases. The C9orf72 repeat expansions were not found in the unaffected familiar member, which partly support co-segregation of this mutation with the disease (Fig. 1).

### Imaging analysis

#### Structural MRI analysis

**Fig. 2:**
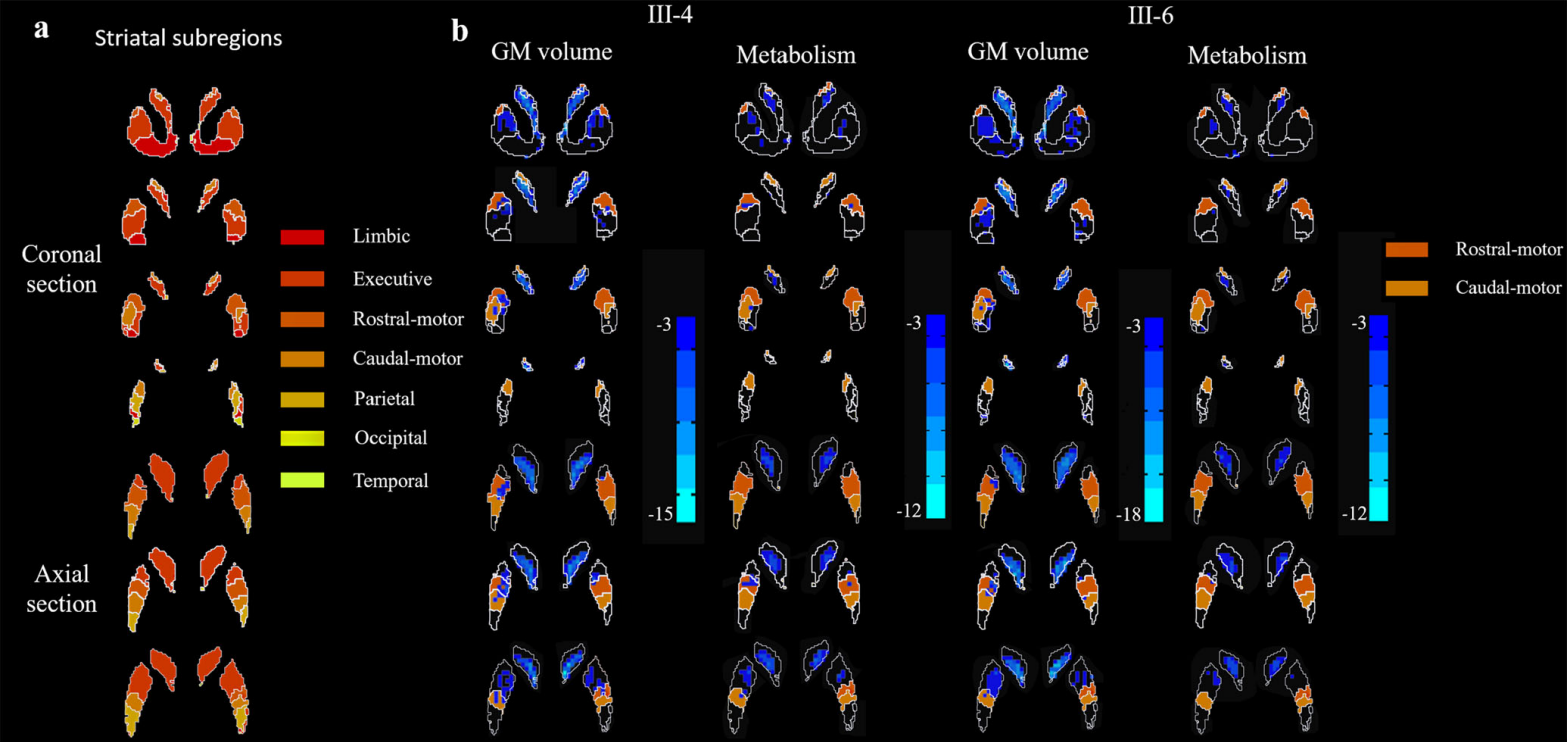
GM atrophy and hypometabolism profiles in functional subregions of the striatum. **a** The striatal parcellations based on intrinsic functional connectivity to the cerebral cortex. Colors indicate functional subdivisions into the limbic, executive, sensorimotor, rostral-motor, caudal-motor, parietal, occipital, and temporal subdivisions. **b** Blue color corresponds to patterns of gray matter loss and hypometabolism in the FTDP patients compared to controls. Results are shown after correction for multiple comparisons using FDR *p* < 0.05. VBM analysis indicated significantly greater atrophy in the rostral-motor and caudal-motor regions of our FTDP patient (III-4) compared to the healthy controls. Similarly, voxel-wise examinations revealed hypometabolism in the rostral-motor and caudal-motor subregions compared to healthy controls, taking age and sex as covariates. In the other patient (III-6), significant GMV loss was also observed in the rostral-motor and caudal-motor subregions. However, no significant hypometabolism was observed in the rostral-motor and caudal-motor subregion.

Bilateral GM volume reductions were significantly more pronounced in the rostral-motor and caudal-motor areas of FTDP patients (III-4 and III-6) than the healthy controls (FDR corrected, *p* < 0.05) (Fig. 2b). Besides motor subdivisions, in patients with FTDP compared with controls, the voxel-wise analysis demonstrated a pattern of decreased GM volume in the limbic, executive, parietal and temporal areas of the striatum (FDR corrected, *p* < 0.05). Detailed data are provided in Supplementary Table 2.

### [^18^F]-FDG PET acquisition and preprocessing

Voxel-wise examinations revealed significantly decreased metabolism within the rostral-motor and caudal-motor regions that mirror areas of GM atrophy in patient III-4 (FDR corrected, *p* < 0.05). This patient had a severe reduction of SUVR in the rostral-motor and caudal-motor subregions, which was 33.9% and 50.7% of the normal, respectively. However, no significant alteration of metabolism was observed in the rostral-motor and caudal-motor regions of the patient III-6. In addition, SUVR analysis revealed limited regions of hypometabolism, which was confined to the limbic, executive, and temporal areas compared to healthy controls (FDR corrected, *p* < 0.05). Regions with significant metabolic changes are shown in Fig. 2b. Detailed data are shown in Table 1.

**Table 1:** Results of the striatal functional distribution in FTDP patients with the C9orf72 Repeat Expansions by T1-MRI, FDG-PET/MRI and DTBZ-PET/CT.

Region	T1-MRI (GM Volume)			FDG-PET (SUVR value)			DTBZ-PET (SUVR value)	
	Control (*n* = 17)	III-4	III-6	Control (*n* = 17)	III-4	III-6	Control (*n* = 6)	III-6
Limbic subregion	0.45 ± 0.04	0.38	0.34^*^	2.07 ± 0.27	1.44^*^	1.33^*^	3.35 ± 0.11	2.30^*^
Executive subregion	0.53 ± 0.04	0.23^*^	0.22^*^	2.16 ± 0.28	0.71^*^	0.81^*^	2.96 ± 0.08	1.68^*^
Rostral-motor subregion	0.48 ± 0.05	0.29^*^	0.38^*^	1.80 ± 0.37	0.64^*^	1.27	2.42 ± 0.10	1.93^*^
Caudal-motor subregion	0.45 ± 0.05	0.27^*^	0.31^*^	1.72 ± 0.40	0.87^*^	1.12	2.83 ± 0.16	1.69^*^
Parietal subregion	0.44 ± 0.05	0.35	0.38	1.57 ± 0.44	1.11	1.57	3.01 ± 0.14	1.88^*^
Occipital subregion	0.40 ± 0.07	0.33	0.28	1.55 ± 0.29	1.67	0.69^*^	2.60 ± 0.31	1.97^*^
Temporal subregion	0.36 ± 0.03	0.24^*^	0.21^*^	1.12 ± 0.24	0.68	0.37^*^	-	-

^*^Indicated values in FTDP patients are 2 SD lower than the mean value for the control group in the same brain regions.

*GM* gray matter, *SUVR* standardized uptake value ratio.

### DTBZ PET analysis

**Fig. 3:**
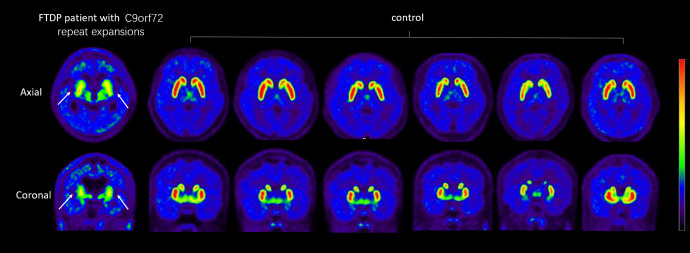
Reduced dopamine terminal in FTPD patients with C9orf72 expansion. DTBZ-PET/CT imaging of the VMAT2 distribution in the FTDP patient (III-6) with C9orf72 expansion and controls (*n* = 6). The same slice is presented for all participants. White arrows indicate obvious dopamine decline of the bilateral striatum in the patient. Note the symmetric decline of vesicular transporter availability without a posterior-to anterior gradient.

By inspection, vesicular monoamine transporter type II (VMAT2) distribution was decreased significantly in the striatum and particularly pronounced in the bilateral caudate nucleus (Fig. 3). ROI analysis showed that the VMAT2 distribution in the motor subregion of the striatum including the rostral-motor and caudal-motor areas, was decreased significantly in the patient III-6 compared with that in the controls (FDR corrected, *p* < 0.05). SUVR analysis suggested that the uptake ratio of VMAT2 decreased to 59.7% of the normal in the caudal-motor region and 80% in the rostral-motor subregion. Detailed data are provided in Table 1.

## Discussion

In this study, we investigated the pathogenesis of parkinsonism associated with striatal motor subdivisions in a pedigree with C9orf72 repeat expansions causing well-characterized FTDP by combining multiple imaging modalities. We believe our results provide a more comprehensive understanding of the mechanism of parkinsonism associated with the striatum in subjects with FTDP who have C9orf72 repeat expansions. The presence of atrophy, hypometabolism, and dopaminergic dysfunction, in the motor subregion of the striatum in FTDP may present a compelling argument in favor of parkinsonism owing to the involvement of motor subregions in the striatum associated with C9orf72 repeat expansions.

We detected alterations at the level of striatal motoric subregions in FTDP patients with C9orf72 repeat expansions and analyzed their roles in parkinsonism. Some previous studies have suggested non-specific subcortical or striatal impairment without any functional distinction^9–11^. It is generally acknowledged that the striatum has connections with the cortex and plays an important role in the striato-cortical circuitry, which has been characterized by several functionally segregated subcircuits that are adjacent, but anatomically different^16^. A connectivity-based (CB) functional striatum atlas, which provides optimal subdivision of the striatum, was used in the present study. Our observations of the atrophy, hypometabolism, and dopamine depletion in the striatal motor part are partly consistent with recent research, in which dopaminergic deficiency in the motor subdivision of the striatum was also found in PD patients^14,17,18^. Therefore, functionally defined striatal motor subregional alterations may provide new perspectives for investigating the pathogenesis of parkinsonism associated with C9orf72 repeat expansions.

Notably, ^18^F-DTBZ PET/CT was used to assess the integrity of the presynaptic dopaminergic pathway in FTDP in our study, because the vesicular monoamine transporter type II (VMAT2) has little metabolism in the body and better stability^19,20^. Moreover, the application of multiple imaging modalities including FDG-PET/MRI and ^18^F-DTBZ PET/CT allowed us to match alterations of structure, metabolism, and dopamine transport function in the same individual, providing a comprehensive illustration of brain changes in patients who have C9orf72 repeat expansions. Our overlapping structural, metabolic and dopaminergic alterations suggest more extensive and symmetric impairment of the striatum comparing to PD patients. The presynaptic dopaminergic synapse selectively degenerated in PD, causing the decrease of dopaminergic uptake with a posterior-to-anterior gradient in the striatum, while the postsynaptic neurons of basal ganglia were less involved in the early phase of Parkinson and the glucose metabolism increased due to compensation^6,7^. On the contrary, as shown in the study, the dopaminergic terminals were disrupted, and the FDG uptake decreased in multiple subregions of the striatum, indicating a perfused presynaptic and postsynaptic interruption. We assume that these different patterns might reflect the primary involvement of striatum result from C9orf72 repeat expansions in FTDP.

Recently, the expanded GGGGCC (G4C2) hexanucleotide repeat in intron 1 of C9orf72 has been confirmed to cause striatal impairment with TDP-43 accumulation in the FTDP patients, which can be clearly distinguished from Parkinson disease-related mechanisms by the absence of α-synuclein-positive Lewy bodies or Lewy neurites in the substantia nigra^9,21,22^. Striatal motor subregions act in conjunction with the cortex through the striato-cortical circuitry, which is associated with pathogenesis of parkinsonism^15,23^. The neuronal degeneration and dopamine depletion of motor subregions may lead to parkinsonism by increased output, which can result in less movement through inhibition of the striato-cortical projection neurons^23^. In our study, all FTDP patients presented with bradykinesia and rigidity, moreover, we found the overlapping structural, metabolic, and dopamine transport functional alterations of the striatal motor subdivisions in in vivo imaging findings, suggesting that TDP-43 pathological deposition may exist in motor subregions to cause functional deterioration. Our findings were partially in line with the only two previous study, which revealed the hypometabolism and dopamine depletion in the striatum as a whole in FTDP patients with C9 repeat expansions^10,24^. These results likely add some support information to our hypothesis wherein C9orf72 repeat expansions may cause the pathological aggregation of TDP-43 in the striatal motor regions, leading to the degeneration and severe loss of dopaminergic neurons, which contribute to parkinsonism in FTDP patients.

Interestingly, in addition to heterogeneity of clinical phenotypes, the FDG-PET results of striatal functional subdivisions provide the first demonstration of heterogeneous neuroimaging phenotypes among familial patients with FTDP who possess the same C9orf72 repeat expansions. The proband (III-4) with parkinsonism as the first symptom showed greater reductions in glucose utilization and GM volume of motor subregions in the striatum. Dysfunction of the striatal motor subdivisions does not seem to depend on the severity of clinical manifestations, but is correlated with initial symptoms or the duration of parkinsonism. Correspondingly, studies have indicated that the degree of dopaminergic deficiency on [^123^I]-FP-CIT SPECT correlated inversely with motor severity in patients with Parkinson’s syndrome^25^. Consistent with the above study, significant dopaminergic deficiency was shown in motor-related subregions of the striatum in patients III-4 with severe parkinsonism. Notably, this patient showed inconsistent results with non-significant hypometabolism and significantly decreased VMAT2 uptake in the striatal motor regions. Similar to previous studies, the dopaminergic PET might more easily identify the striatal dysfunction associated with parkinsonism compared with FDG-PET^10,26^. Given the limited evidence, further investigation of striatal motor subdivisions in larger FTDP cohorts with C9orf72 repeat expansions need to be undertaken. Nevertheless, these results indicate that parkinsonism might be present in the pure FTDP phenotype with C9orf72 repeat expansion in China, thus suggesting that more attention should be paid to parkinsonism in patients with C9orf72 repeat expansion in clinical practice. Furthermore, measuring structure, metabolism, and dopaminergic changes in a functionally defined motor-related striatal region may provide a more sensitive tool to detect C9orf72-associated specific changes of the striatum, and could thus improve clinical decision and individual prognosis.

This study had some limitations. First, although patients belonged to the same family, the number of patients is limited due to the rarity of FTDP harboring C9orf72 repeat expansions, which raises limitations in data interpretation. Therefore, our imaging findings are exploratory; additional studies are needed to verify our hypothesis and confirm our present findings in larger cohorts. Second, long-term follow-up should be conducted to observe the alteration in the motor subregion of the striatum, to deeply understand its contributions in the pathogenesis of parkinsonism. In addition, because of less attention and difficulty in early recognition, the enrolled FTDP patients were in a relatively advanced stage of the disease with severe clinical manifestations, whereas patients in the early stage of the disease, especially asymptomatic carriers in this family were not included. For future studies, we aim to include asymptomatic carriers and collect additional data of longitudinal studies to further understand the pathogenesis and underlying mechanisms of FTDP.

In conclusion, our findings might expand the understanding pathophysiological mechanism underlying parkinsonism in FTDP with C9orf72 repeat expansions. The degeneration of the rostral-motor and caudal-motor subdivisions functionally might impair the integrity of dopaminergic terminals in the striatum, disturbing the efficient interplay between motor processing areas and impairing motor control in patients with FTDP harboring C9orf72 repeat expansions. However, we regard our data primarily as a foundation for further studies and believe that the findings presented here should be validated in larger cohorts.

## Methods

### Patients and controls

Nineteen participants took part in this study, which was approved by the Ethics Committees of the Xuanwu Hospital of Capital Medical University, China, and was conducted in accordance with the principles stated in the Declaration of Helsinki. Written informed consent was obtained from all the participants or legal guardians.

The pedigree comprised three affected individuals (two alive) in two generations (Fig. 1). Both patients underwent detailed clinical interviews, physical examinations, the Unified Parkinson’s Disease Rating Scale (UPDRS) Part III (which assesses motor function), genetic testing, and cerebral 18F-fluorodeoxyglucose positron emission tomography/magnetic resonance imaging examinations (18F-FDG PET/MRI), and one of the patients (III-6) underwent [18 F]-dihydrotetrabenazine positron emission tomography/computed tomography ([18 F]-DTBZ PET/CT) within two months of recruitment (three years after the onset of symptoms). In addition, neuropsychological assessments were performed in the proband (III-4), but failed in her brother (III-6). The diagnosis was performed according to the consensus criteria for probable behavioral variant FTD (bvFTD) published in 2011^27^. Clinically, parkinsonism is identified when at least two clinical features among bradykinesia, rigidity, resting tremor and postural instability are present, without a history of known causative factors such as repeated strokes, encephalitis, or neuroleptic treatment, and without supranuclear gaze abnormalities and ataxia. Finally, two symptomatic living patients (III-4, III-6) from this pedigree were diagnosed with FTDP by a multidisciplinary team including three neurologists (L.Y.W., S.Y.L., and J.H.M), a neurogeneticist (P.C.) and a neuroimaging specialist (H.W.Q). Meanwhile, healthy controls (*n* = 17) were enrolled and underwent clinical interviews and examination, including neuropsychological assessments, genetic testing, cerebral [^18^F]-FDG PET/MRI and [^18^F]-DTBZ PET/CT.

### Genetic analyses

We extracted genomic DNA from fresh peripheral blood leukocytes and used an Agilent SureSelect Human All Exon V6 Kit (Agilent Technologies, Santa Clara, CA, USA) to generate a sequencing library for whole exome sequencing (WES). The prepared libraries were sequenced using the HiSeq-2000 platform (Illumina, San Diego, CA, USA). DNA sequencing and genetic analysis were performed as previously described^5^. Repeat primed PCR was performed as previously described to obtain a qualitative estimation of the presence of C9orf72-expanded repeats^28^.

We investigated variants from genes associated with ‘dementia and parkinsonism’ according to several databases: the Human Gene Mutation Database (HGMD, http://www.hgmd.cf.ac.uk/ac/search.php), Online Mendelian Inheritance in Man (OMIM; https://www.omim.org/), Clinvar (https://www.ncbi.nlm.nih.gov/clinvar), and GeneCards (https://www.genecards.org). All shortlisted genes that were associated with dementia and parkinsonism were then verified by UniProt (http://www.uniprot.org/), and the selected genes were further confirmed by MalaCards (http://www.malacards.org/). Our final analysis included 69 genes that were associated with FTD, parkinsonism, and other neurodegenerative diseases. Supplementary Table 3 provides further details of the genes selected for analysis.

### Neuroimaging acquisition and preprocessing

#### Structural MRI acquisition and preprocessing

All subjects underwent a hybrid 3.0 T TOF PET/MR (SIGNA PET/MR, GE Healthcare, WI, USA).

A 3D T1-weighted images were acquired using the scanning parameters of repetition time (TR) = 6.9 ms, echo time (TE) = 2.98 ms, flip angle = 12°, inversion time = 450 ms, matrix size = 256 × 256, field of view = 256 × 256 mm^2^, slice thickness = 1 mm, 192 sagittal slices with no gap, voxel size = 1 × 1 × 1 mm^3^, and acquisition time = 4 min 48 s. Patterns of cerebral atrophy were assessed using voxel-based morphometry (VBM) based on the Computational Anatomy Toolbox (CAT12) toolbox segment data pipeline implemented within Statistical Parametric Mapping 12(SPM12, http://www.fil.ion.ucl.ac.uk/spm).

### [^18^F]-FDG PET acquisition and preprocessing

The [^18^F]-FDG PET images were acquired according to standard protocols and following the European Association of Nuclear Medicine (EANM) guidelines^29^. The parameters of 18F-FDG-PET data were as follows: matrix size = 192 × 192, field of view = 350 × 350 mm^2^, and pixel size = 1.82 × 1.82 × 2.78 mm^3^ and included corrections for random coincidences, dead time, scatter, and photon attenuation. The [18 F]-FDG PET image processing and analyses were performed by means of SPM12 implemented in MATLAB software (Mathwork, Inc., Natick, MA, USA). The FDG-PET scan intensity was normalized using a whole cerebellum reference region to create standardized uptake value ratio (SUVR) images.

### DTBZ PET acquisition and preprocessing

The [^18^F]- dihydrotetrabenazine radiotracer (^18^F-DTBZ) was prepared from aqueous [^18^F]-fluoride. An average frame was acquired over an approximately 15-min period, 90 min after the injection of approximately 250 MBq of ^18^F-DTBZ. Images were reconstructed using 3D iterative method. The frames were spatially segmented, co-registered and normalized for each subject using the PNEURO utilities suite. Regional SUVRs were calculated for each participant using the occipital cortex as reference^19^.

### Analysis at the striatal subregion level

Instead of using arbitrary anatomical landmarks to subdivide the striatum, we used the substriatal ROI from the Oxford-GSK-Imanova Striatal Connectivity Atlas^15^, a probabilistic atlas of substriatal regions segmented according to their white-matter connectivity to cortical regions. This atlas subdivides the striatum into seven subregions based on their differential cortical connectivity patterns (Fig. 2a). Thus, the limbic striatal subregion is connected with the orbital gyrus, gyrus rectus, and subcallosal gyrus/ventral anterior cingulate; the executive subregion is connected with the rostral superior and middle frontal gyri and the dorsal prefrontal cortex; the rostral-motor subregion is connected with the connectivity to rostral area 6, pre-supplementary motor area, and the frontal eye field region; the caudal-motor subregion is connected with the precentral gyrus; and a parietal, occipital, and temporal subregions are connected with the parietal, occipital, and temporal lobes, respectively.

Voxel-wise two-sample t-tests were used to compare regional GM volume and glucose metabolism between the FTDP patients and controls^30,31^. GM volume and SUVR data acquired from the controls were used as a normal database. To evaluate individual changes of GM volume and SUVR, a two-sample *t* test was performed between individual patient data and the normal database; age and gender were treated as covariates to reduce the potential effects of these variables. Considering the sample size and unbalanced numbers of patients in the two groups, we used the (FDR) procedure to control the expected proportion of false positive voxels at p < 0.05. Atlas-based ROI analysis of the structural MRI and PET images was performed to extract SUVR from the Oxford-GSK-Imanova Striatal Connectivity atlas.

### Statistical analyses

Statistical analyses were carried out in SPSS 22.0 (IBM Corporation, Armonk, NY, USA). Continuous data are represented as means ± standard deviations. Dichotomous data are represented as absolute values. Group differences were tested using Student’s *t* test for continuous data and chi-square and Fisher’s exact tests for categorical data. Statistical significance was set at *p* < 0.05.

## Supplementary information

Supplementary information

## Data Availability

The data that support the findings of this study are available from the corresponding author upon reasonable request. This study’s DNA sequencing data have been deposited in the Genome Sequence Archive (Genomics, Proteomics & Bioinformatics 2021) in National Genomics Data Center (Nucleic Acids Res 2022), China National Center for Bioinformation/Beijing Institute of Genomics, Chinese Academy of Sciences (GSA-Human: HRA002999) that are publicly accessible at https://ngdc.cncb.ac.cn/gsa-human.

## Code availability

All codes used in this study are available from the corresponding author upon reasonable request.
