# Supplementary material for: Involvement of striatal motoric subregions in familial frontotemporal dementia with parkinsonism harboring the C9orf72 repeat expansions
Source: NPJ Parkinsons Dis. 2022 Oct 6;8:128. doi: 10.1038/s41531-022-00398-5 (PMC9537191; doi:10.1038/s41531-022-00398-5)
Supplement: Supplementary file 1 — Supplementary information [file 41531_2022_398_MOESM1_ESM.pdf]

Supplementary Table 1. Clinical, neuropsychological, and neuroimaging findings of these two FTDP patients

	Patient 1 (III-4)	Patient 2 (III-6)
Age at evaluation (y)	68	65
Gender	Female	Male
Education	19	12
Age at onset	65	62
Family history	+	+
Parkinsonism	+	+
Bradykinesia	+	+
Rigidity	+	+
Tremor	-	-
Postural instability	-	-
Response to levodopa	+, Partial	-
FTD		
Dementia	+	+
Personality changes	+	+
Speech changes	+	+
Physical examination		
Eye movements	-	-
Babinski reflex	-	-
Muscle atrophy	-	-
Fasciculations	-	-
Neuropsychology assessment		
MMSE	13	NA
MoCA	5	NA
AVLT: immediate recall	3	NA
AVLT: delayed recall	0	NA
Trial making test A (s)	NA	NA
Trial making test B (s)	NA	NA
BNT	12	NA
CDR	2	2
NPI-Q	16	9
FBI	25	23
MBI-C	18	19
Neuroimaging examination		
Brain atrophy on MRI	+	+
[18F]-FDG PET/MRI	+	+
[18F]-DTBZ PET/CT	NA	+

Supplementary Table 2. Regions with grey atrophy and reduced glucose metabolism in FTDP patients compared to controls significant at the cluster level by FDR corrected for multiple comparisons over the functional distribution of the striatum.

Patient	Region	Side	Cluster Size	T value	
				Peak intensity	Mean
**III-4**	**GM atrophy**				
	Limbic	R	16	-12.39	-5.18
	Limbic	L	15	-9.25	-5.60
	Executive	R	253	-15.00	-5.97
	Executive	L	258	-14.12	-6.18
	Rostral-motor	R	9	-6.02	-4.02
	Rostral-motor	L	19	-7.08	-3.92
	Caudal-motor	R	27	-11.47	-5.33
	Caudal-motor	L	14	-8.02	-4.03
	Parietal	R	12	-9.65	-4.89
	Parietal	L	1	-8.45	-8.45
	Temporal	R	1	-11.61	-11.61
	**GM hypometabolism**				
	Limbic	R	44	-11.09	-5.16
	Limbic	L	52	-9.72	-5.03
	Executive	R	160	-11.03	-5.54
	Executive	L	203	-11.79	-5.65
	Rostral-motor	L	6	-4.71	-3.93
	Caudal-motor	R	1	-3.42	-3.42
	Caudal-motor	L	2	-3.46	-3.30
	Temporal	L	1	-3.11	-3.11
**III-6**	**GM atrophy**				
	Limbic	R	51	-14.11	-5.42
	Limbic	L	55	-14.41	-4.88
	Executive	R	296	-16.37	-6.80
	Executive	L	351	-16.11	-6.59
	Rostral-motor	R	5	-6.65	-4.07
	Rostral-motor	L	13	-5.59	-3.90
	Caudal-motor	R	27	-12.81	-5.64
	Caudal-motor	L	13	-8.43	-4.10
	Parietal	R	15	-10.70	-4.88
	Parietal	L	5	-9.54	-4.46
	Temporal	R	1	-12.09	-12.09
	Temporal	L	1	-6.53	-6.53
	**GM hypometabolism**				
	Limbic	R	30	-11.68	-5.52
	Limbic	L	25	-10.71	-5.44
	Executive	R	129	-11.14	-5.60
	Executive	L	176	-10.89	-5.64

Supplementary Table 3. The selective gene associated with FTD, parkinsonism and other neurodegenerative diseases for analyses

Classification	Gene list
Frontotemporal Dementia-associated gene	MAPT, GRN, TARDBP, FUS, VCP, CHMP2B, SQSTM1, CHCHD10, TBK1, UBQLN2, OPTN, CCNF, TIA1
Parkinsonism -associated gene	ACMSD, ADH1C, ATP1A3, ATP6AP2, ATXN8OS, CHCHD2, DNAJC13, DNAJC6, FBXO7, GIGYF2, GLUD2, HTRA2, LRP10, LRRK2, PACRG, PARK7, PINK1, PLA2G6, PRKN,<br>RAB29, SLC18A2, SLC6A3, SNCA SNCAIP, SYNJ1, TAF1, TBP, UCHL1, UQCRC1, VPS13C, VPS35, VPS35L, GBA, EIF4G1
Other neurodegeneration-associated gene	PSEN1, PSEN2, APP, TREM2, DCTN1, SIGMAR1, hnRNPA2B1, NOTCH3, TRPM7, ABCC1, APBB2, ATP13A2, SPG21, DMT1, VPS2B, ALS17, SCN8A, COQ2, TSC1, TSC2, HCFC1, ITPR3
